# Citrus tristeza virus-host interactions

**DOI:** 10.3389/fmicb.2013.00088

**Published:** 2013-05-14

**Authors:** W. O. Dawson, S. M. Garnsey, S. Tatineni, S. Y. Folimonova, S. J. Harper, S. Gowda

**Affiliations:** ^1^Department of Plant Pathology, Citrus Research and Education Center, University of FloridaLake Alfred, FL, USA; ^2^Department of Plant Pathology, University of FloridaGainesville, FL, USA

**Keywords:** Citrus tristeza virus, citrus, disease, host-interactions, stem pitting, seedling yellows

## Abstract

Citrus tristeza virus (CTV) is a phloem-limited virus whose natural host range is restricted to citrus and related species. Although the virus has killed millions of trees, almost destroying whole industries, and continually limits production in many citrus growing areas, most isolates are mild or symptomless in most of their host range. There is little understanding of how the virus causes severe disease in some citrus and none in others. Movement and distribution of CTV differs considerably from that of well-studied viruses of herbaceous plants where movement occurs largely through adjacent cells. In contrast, CTV systemically infects plants mainly by long-distance movement with only limited cell-to-cell movement. The virus is transported through sieve elements and occasionally enters an adjacent companion or phloem parenchyma cell where virus replication occurs. In some plants this is followed by cell-to-cell movement into only a small cluster of adjacent cells, while in others there is no cell-to-cell movement. Different proportions of cells adjacent to sieve elements become infected in different plant species. This appears to be related to how well viral gene products interact with specific hosts. CTV has three genes (p33, p18, and p13) that are not necessary for infection of most of its hosts, but are needed in different combinations for infection of certain citrus species. These genes apparently were acquired by the virus to extend its host range. Some specific viral gene products have been implicated in symptom induction. Remarkably, the deletion of these genes from the virus genome can induce large increases in stem pitting (SP) symptoms. The p23 gene, which is a suppressor of RNA silencing and a regulator of viral RNA synthesis, has been shown to be the cause of seedling yellows (SY) symptoms in sour orange. Most isolates of CTV in nature are populations of different strains of CTV. The next frontier of CTV biology is the understanding how the virus variants in those mixtures interact with each other and cause diseases.

## Introduction

Plant viruses are parasites that multiply and survive in plants. Their genomes are too small to effect their own replication and movement throughout plants alone. They must utilize a combination of virus-encoded genes working complementarily with host genes. Thus, viruses have evolved specific genes whose products interact with the host to replicate the virus, other viral gene products to interact with host to allow accumulation and distribution throughout the host plants, and other gene products to interact with vectors to allow transmission to other plants. Viral genes that are involved in replication tend to be conserved, suggesting that replication within a plant cell is rather generic. Indeed, many viruses are able to replicate in protoplasts from plants in which they are unable to systemically invade. In contrast, viral genes involved in spread within plants tend to be much less conserved. This observation suggests that different viruses use different strategies for invading their hosts. Members of the *Closteroviridae*, which consists of *Closterovirus, Crinivirus*, and *Ampelovirus* genera with mono-, bi-, or tripartite genomes, provide some of the better examples of combinations of conserved and unique genes. They all encode a mixture of conserved signature gene modules along with unique genes with no relationship found in other members of the family. The conserved gene products are involved primarily in replication and virion assembly. In fact, some domains and *cis*-acting elements involved in replication can be exchanged between different viruses. Additionally, members within a genus possess 1–5 unique genes. These gene products are thought to have evolved to interact exclusively with their specific hosts (Karasev, 2000; Dolja et al., 2006).

There are several unique features of the *Closterviridae*. First is that they have morphologically polar virions (Agranovsky et al., 1995; Febres et al., 1996; Tian et al., 1999), which is unique to this virus group. The second feature is that they encode proteins with similarities to molecular chaperones that are required for assembly (Peremyslov et al., 1999; Alzhanova et al., 2001) and possibly insect interactions (Tian et al., 1999). However, the most significant feature is that these viruses have evolved to be transmitted similarly, in a semi-persistent manner, but by at least three different types of insect vectors: aphids, whiteflies, and mealybugs. Based on sequence comparisons, they have two conserved gene modules. The first consists of replicase-associated genes including one or two protease (PRO) domains plus methyltransferase- (MT) and helicase- (HEL) like domains and an RNA-dependent RNA polymerase (POL) domain, with the latter being translated by a +1 frame-shift. Although the order of these domains and the large intragenic regions are characteristic of this group of viruses, similar domains occur in most RNA viruses. These gene products are produced from the genomic RNA. The 3′ genes are expressed through subgenomic (sg) RNAs. The second signature gene module consists of five or six genes that encode the major coat protein (CP) and a related minor coat protein (CPm) that varies in size and genomic position among the different viruses plus three other proteins: a protein closely related to the ubiquitous HSP70 proteins (Karasev et al., 1992; Agranovsky et al., 1997), a small (6 kDa) hydrophobic protein proposed as a membrane anchor, and a protein of ~60 kDa. As noted above, these viruses contain 1–5 non-conserved genes with no relationship to each other.

**Figure 1:**
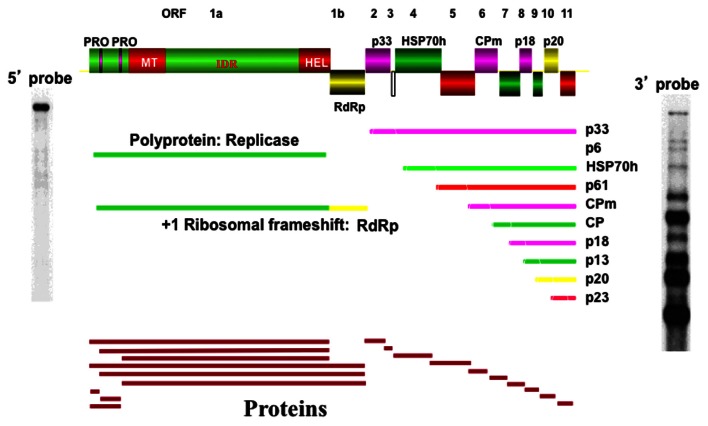
**A schematic diagram of the genetic organization of CTV showing ORFs (open boxes) of each gene.** PRO, papain-like proteases; MT, methyl transferase-like domain; IDR, large interdomain region; HEL, helicase-like domain; RdRp, RNA-dependent RNA polymerase domain; HSP70h, analog to heat shock protein; CPm and CP, minor and major coat proteins. Below are expected protein products and the 10 subgenomic RNAs (the ORF translated is shown in parentheses). Left and Right are Northern hybridization analyses of total RNAs hybridized using a 5′-specific probe (left) or a 3′-specific probe (right).

Citrus tristeza virus (CTV) has a 19.3-kb single-stranded positive-sense RNA genome (Bar-Joseph et al., 1979; Pappu et al., 1994; Karasev et al., 1995). The genomic RNA of CTV is organized into 12 open reading frames (ORFs), which potentially encode at least 19 final proteins (Karasev, 2000). Ten 3′ genes are expressed through a nested set of 3′ co-terminal sg mRNAs (Hilf et al., 1995), which consist of the signature ORFs (Pappu et al., 1994) plus 5 non-conserved genes (Figure 1).

## Requirements for replication

The ten 3′ genes are not required for replication of the genomic RNA (Satyanarayana et al., 1999). A replicon with only ORFs 1a and 1b plus the 5′ and 3′ non-translated regions (NTR) replicates well in protoplasts (Satyanarayana et al., 1999). The 5′ NTR is 107 nts and contains a precise structure with two stem loops. This was first noticed when López et al. (1998) analyzed the 5′ sequences of nine different CTV isolates that varied as much as 58%, yet all folded into the same structure. Gowda et al. (2003) found that the precise stem-loop secondary structures, in contrast to the primary sequence, are necessary for replication. In contrast to most other RNA plant viruses, the 3′ NTR does not contain a poly-A tract nor does it appear to fold as a tRNA mimic. Instead it is highly conserved among different CTV strains and is predicted to consist of 10 stem-loop structures with the replication signals within the 3′ 234 nts (Satyanarayana et al., 2002a). One of the 3′ genes, p23, although not essential, greatly affects the plus-strand to minus-strand ratio of CTV RNAs (Satyanarayana et al., 2002b). Mutants without a functional p23 gene produce almost equal amounts of negative and positive strands. The wild-type virus produces plus-stranded genomic and sgRNAs ~10–50 times more than minus strands. The absence of a functional p23 gene also reduces or prevents protein production from 3′ genes apparently by preventing the production of single-stranded RNAs to serve as messenger RNAs.

## Requirements for assembly

Although CTV virions had been semi-purified and characterized, only much later was it found that virions consisted of two coat proteins (Bar-Joseph et al., 1979; Agranovsky et al., 1995; Febres et al., 1996). Most of the virion is encapsidated by coat CP, but ~3% of the virion from the 5′ end is encapsidated by the minor coat CPm (Satyanarayana et al., 2004). Besides CP and CPm, the HSP70 homolog (p65) and p61 are involved in assembly of virions (Satyanarayana et al., 2000). Assembly of CPm is initiated at the stem-loop structures in the 5′ NTR and in the presence of HSP70h and p61 encapsidation stops at approximately nt 630 (Gowda et al., 2003; Satyanarayana et al., 2004). In the absence of HSP70h and p61, encapsidation occurs much more slowly and continues toward the 3′ terminus (Satyanarayana et al., 2004). Neither protein is active alone. Thus, these two proteins in combination enhance encapsidation by CPm and limit it to the 5′ end of the genomic RNA (Satyanarayana et al., 2004). Additionally, encapsidation by CPm in the absence of other assembly related proteins shows remarkably high specificity (Tatineni et al., 2010). Heterologous CPm's with 95–96% amino acid identity from related strains substituted into a CTV replicon with CPm as the only assembly related ORF, generally failed to initiate encapsidation. However, the heterologous CPm in combination with both HSP70h and p61 proteins, but not HSP70h or p61alone, encapsidated at wild-type levels, suggesting that non-specific interaction of CPm and its origin of assembly was mitigated by the combination of HSP70h and p61. Thus, in addition to enhanced virion formation and restriction of CPm encapsidation to the 5′ 630 nts of the genomic RNA, the HSP70h and p61 proteins facilitate encapsidation by heterologous CPm's.

## Movement in citrus hosts

To establish a productive infection in a host a plant virus needs to be able to move throughout a plant from an initially infected cell. Success depends upon compatible interactions between viral and host factors. Generally, systemic movement is thought to involve two distinct processes: cell-to-cell movement, which is a process that allows the virus to transverse the cell wall between adjacent cells, and long-distance movement, which is a process that allows the virus to enter the sieve element from an adjacent nucleated cell and rapidly move through the connected sieve elements, followed by its exit into another adjacent phloem-associated cell at a distal region of the plant. A major obstacle for the spreading virus is to cross the boundaries represented by the cell wall. For this purpose most viruses utilize specific virus-encoded movement proteins as well as some host proteins that facilitate their translocation through plasmodesmata channels. The viral proteins and their interactions with the host during cell-to-cell movement are fairly well-known (reviewed in Waigmann et al., 2004; Scholthof, 2005; Lucas, 2006). However, the mechanisms of long-distance transport and factors that aid virus entrance into phloem tissue, further vascular movement, and unloading from phloem are much less understood.

**Figure 2:**
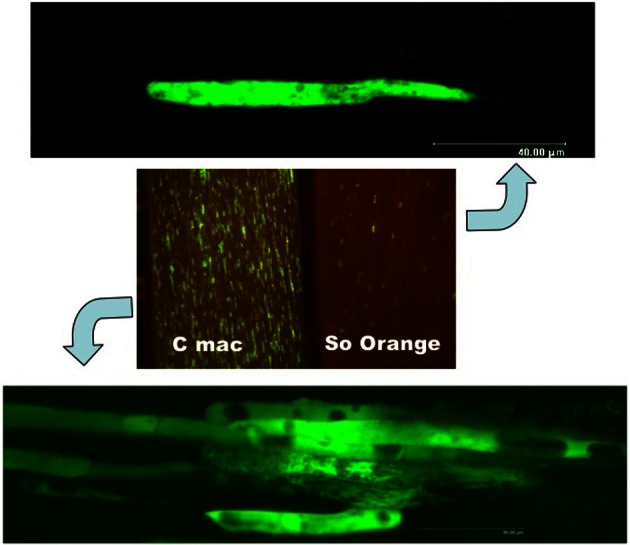
**Detection of GFP fluorescence in phloem-associated cells of *Citrus macrophylla* (C mac) and sour orange (So Orange) under a fluorescence-dissecting microscope (center) or a confocal laser scanning microscope showing single cell infections (top) and multiple cell infections (bottom)**.

CTV generally follows the patterns described above, but the degrees of both cell-to-cell and long-distance movement are more limited than in most well-described systems, and this limitation varies depending on the citrus host. Since CTV infections are limited to phloem-associated cells, the infection can be most easily viewed by looking at fluorescence from green fluorescent protein (GFP)-tagged CTV in peeled bark that exposes phloem cells. In all citrus hosts, long-distance movement appears to be limited to relatively few initial infection sites. In the more susceptible hosts, *C. macrophylla* and Mexican lime, we estimated that only about 10–20% of the phloem-associated cells were infected (Folimonova et al., 2008). The number of fluorescent cells in grapefruit and sour orange bark patches was much less, with sweet orange being intermediate. Also, there was a difference in the size of the fluorescent areas. In the more susceptible species, *C. macrophylla* and Mexican lime, infection sites consisted of clusters of 3–12 cells. In the less susceptible species, sour orange, there were fewer infection sites and they usually were single cells (Figure 2). Sweet orange again tended to be intermediate between these two extremes. Our interpretation is that systemic invasion of CTV begins when the virus enters sieve elements of the phloem, which transport the virus from some distal position in the direction of sugar movement (source to sink), after which at some point the virus exits into an adjacent cell, usually in stems and leaf veins of a new flush. We assume that the adjacent cell is a companion or phloem parenchyma cell, but this differentiation in citrus phloem is not readily apparent, especially when using confocal microscopy of GFP-labeled virus. We refer to this process as “long-distance” movement. We consider the movement of virus to adjacent cells to fill the clusters of multiple cells as “cell-to-cell” movement. Apparently both long-distance and cell-to-cell movement mechanisms of CTV work differently in different citrus species.

In the more susceptible citrus species, CTV also has limited cell-to-cell movement that produces small clusters of infected cells. However, in less susceptible citrus species, it appears that little or no cell-to-cell movement occurs. The virus is able to exit sieve elements but cannot spread to adjacent cells, resulting in infection of isolated single cells. Thus, CTV provides a new pattern of systemic infection in which the virus appears to function with only the long-distance movement mechanism, yet is able to survive in nature. Such a movement pattern has not been described previously. It is not known whether this pattern is characteristic of other members of the *Closterovirdae* or other phloem-limited viruses.

## Aphid transmission

CTV generally has been moved long distances into new areas by transport of infected planting (or propagating) materials. Prior to the advent of rapid shipping in the nineteenth century, importation of citrus occurred only as seed, avoiding CTV spread as the virus is not transmissible by seed. However, as navigation improved, citrus was moved as plants or budwood, and so was CTV. Presently, the problem is that since even severe isolates are symptomless in some of their hosts, the virus often is spread by well-meaning individuals moving an infected but non-symptomatic plant or budwood from such a plant into a new area. Afterwards, local spread is by aphids, where transmission is in a semipersistent manner. This combination has effectively spread CTV (Moreno et al., 2008).

Factors affecting aphid transmission include isolate or strain differences of the virus, the aphid species, plant donor and receptor varieties, the environmental conditions, and the number of aphids involved (Roistacher and Moreno, 1990). In addition, specific isolates or strains of CTV in mixtures may not be equally distributed throughout the source plant, further reducing the likelihood of successful transmission (D'Urso et al., 2000). Finally, aphids show a marked preference for some citrus species over others, for example it has been observed in feeding choice experiments that *Aphis gossypii* preferentially infests mandarins or sweet oranges over lemons (Roistacher et al., 1984). Similarly, *A. gossypii* exhibited longer feeding periods on Mexican limes than sweet oranges (Backus and Bennett, 2009), suggesting that host preference can also affect transmission efficiency (Roistacher and Bar-Joseph, 1984; Hermoso-de-Mendoza et al., 1988; Cambra et al., 2000).

In addition, the observed movement and distribution of CTV correspond with observations of aphid transmissibility from and to specific citrus species. As mentioned earlier, there is a gradient of infection in citrus species, from frequent clusters of infected cells present in *C. macrophylla* to a scattered distribution of single cells in grapefruit and sour orange. By extrapolation one may suggest the scattered distribution of CTV in the latter species reduces the probability of virus acquisition by the aphid, and the lower titer reduces the chance of successful infection, which explains reports of grapefruit, sweet lime, sour orange, and lemon being both poor donor and receptor hosts (Bar-Joseph et al., 1977; Roistacher and Bar-Joseph, 1984; Hermoso-de-Mendoza et al., 1988). These differences in aphid transmission rates may have epidemiological consequences in the field (Moreno et al., 1988; Gottwald et al., 1996).

## Silencing of RNAi

Not only must the virus have the capacity to produce proteins that interact with the host to allow cell-to-cell and long-distance movement, it must also have the ability to escape from the host's surveillance system. Plants have evolved an RNA silencing process, one function of which is to protect them against viruses (Dunoyer and Voinnet, 2005; Wang and Metzlaff, 2005). Viruses generally produce double-stranded RNA sequences that are subject to degradation resulting in production of small RNAs that, in turn, target the homologous sequences in the viral RNA, thus preventing systemic infection. Sometimes the result is a “recovery” phenotype. In turn, viruses generally encode proteins referred to as silencing suppressors that counteract the RNAi plant defense system to allow a systemic infection to be established and maintained (Voinnet et al., 1999; Roth et al., 2004; Qu and Morris, 2005). Mutations of viral suppressor genes generally result in reduction or prevention of systemic infection (Chu et al., 2000; Qu and Morris, 2002).

Citrus species utilize RNAi to reduce CTV titer and slow the progress of systemic infection. Thus, as with other viruses, over the course of its evolutionary history, CTV has acquired or adapted genes that exhibit suppression of silencing, namely p20, p23, and CP (Lu et al., 2004). The CP and p20 gene products function to suppress intercellular silencing, preventing the spread of the silencing signal, and it is presumed, activation of host defenses, while p20 and p23 suppress intracellular silencing and reduce viral degradation. Transgenic expression of p23 has been reported to increase the number and size of infection foci and thus the CTV titer in sour orange, and to release CTV from strict phloem-limitation in sour and sweet orange plants (Fagoaga et al., 2011). The p23 and CP genes also have additional roles in the viral replication cycle, respectively, control of negative strand accumulation and encapsidation. Even when the virus establishes a systemic infection, some degree of silencing and degradation of the CTV genome occurs, regardless of host species or viral strain (Ruiz-Ruiz et al., 2011; Harper, unpublished), which raises an important point to be made that host RNAi cannot completely inhibit or eliminate viral replication or infection, and the three suppressors of silencing cannot completely block the RNAi pathway. From an evolutionary perspective this competition has been likened to an “arms race” (Obbard et al., 2009), and although one would expect the rapidly evolving virus to overcome host RNAi, stabilizing selection may prevent further adaptation, and complete shutdown of the host RNAi pathway would prevent host-cell regulation, leading to severe symptoms and/or death of the plant.

## Some genes are not needed for some hosts

CTV contains five genes, p33, p18, p13, p20, and p23, in the 3′ half of the genome, which are not related to genes of other members of the *Closteroviridae*. We examined whether these genes are necessary for systemic infection of citrus trees by deleting single genes one at a time (Tatineni et al., 2008). The deletion of p20 or p23 prevented systemic infection. Apparently both are needed for counter action against the host RNAi resistance mechanism. Additionally, p23 affects replication of CTV RNA (Satyanarayana et al., 2002b).

However, we found that deletions within the p33, p18, or p13 ORFs individually resulted in no significant loss of ability of the virus to infect, multiply, and spread throughout our common laboratory hosts, *C. macrophylla* and Mexican lime (Tatineni et al., 2008). Furthermore, deletions in the p33, p18, and p13 genes in all possible combinations including deletions in all three genes allowed the virus to systemically invade these plants. GFP-tagged CTV with deletions in the p33 ORF or the p33, p18, and p13 ORFs demonstrated that the movement and distribution of these deletion mutants were similar to those of the wild-type virus.

Because CTV was able to move in these hosts by both cell-to-cell and long-distance movement, it is expected that the virus has other genes that function as a minimal set of movement genes for these hosts. Yet, it was not expected that the virus would retain genes that it did not need. We further examined the roles of these expendable genes (p33, p18, and p13) in a wider range of citrus species and relatives within the CTV host range and found that they are needed for systemic infection of some of the hosts (Tatineni et al., 2011). However, different genes were required for systemic infection of different hosts. The p33 gene was required for systemic infection of sour orange and lemon trees. It would appear that the p33 is involved in interactions with host proteins of sour orange and lemon for successful long-distance transport of CTV. Either the p33 or the p18 gene was sufficient for systemic infection of grapefruit trees. Deletion of both genes prevented systemic infection, but deletion of either one did not. These results suggest that the p33 and p18 gene products provide similar or redundant functions in grapefruit. Similarly, the p33 or the p13 gene was sufficient for systemic infection of calamondin plants, again suggesting that these two gene products provide similar or redundant functions in this host. This property of either of two different genes providing the same function appears to be a rare property for viruses.

Thus, these three genes are required for systemic infection by CTV of its full host range, but different genes are specific for different hosts (Tatineni et al., 2011). These findings suggest that CTV acquired multiple non-conserved genes for movement and overcoming host resistance and some of these genes (p33, p18, and p13) were gained to extend its host range further.

## Induction of disease symptoms by CTV

Although viruses of plants have been focused upon because of the diseases they cause, the ultimate interaction when a virus evolves with a host is likely “no disease” or “limited disease.” Yet, as viruses interact with plant hosts, they do sometimes cause disease. When disease occurs in a plant, it is often accidental due to the virus moving to a new host presented to it by agricultural practices. Disease symptoms usually occur on portions of the plant that develop and grow subsequent to viral infection. Rarely do symptoms occur in areas of the plant that are fully developed at the time of infection. Disease often results from interference with differentiation or development. Yet, when diseases do occur, they can cause severe damage to plants, and in agricultural crops diseases cause economic losses, sometimes even preventing some crops from being grown.

Examination of a large number of virus isolates (which can be populations of different strains) on a series of different plants from the host range suggested that CTV has the largest number of distinct phenotypes of any plant viruses (Garnsey et al., 2005; Hilf et al., 2005; Moreno et al., 2008). The number of phenotypes is amplified by the specificity of the phenotypes in different plants. For example, some isolates cause specific symptoms in grapefruit but not other varieties, some in sweet orange and not other varieties, some in both and some in neither. This level of specificity occurs across the whole host range. Besides these disease symptoms seen in the field, vein clearing, leaf cupping, and temporary yellowing and stunting of young seedlings are phenotypes used in greenhouse diagnosis. Yet, it should be kept in mind that the most frequent phenotype is no symptoms.

However, CTV does cause or threaten to cause serious economic damage to all citrus industries. Depending on the virus isolate and the variety/rootstock combination, CTV can cause any of four distinct syndromes (Bar-Joseph et al., 1989; Bar-Joseph and Dawson, 2008; Moreno et al., 2008). “Decline” results in death of sweet orange, mandarin, or grapefruit varieties on sour orange rootstocks. During the last century, CTV-induced decline destroyed entire citrus industries worldwide, leading to the substitution of the most desirable sour orange rootstock by other rootstocks that are tolerant to CTV decline, but that are inferior for tree growth and fruit production in saline or alkaline soils, and also more susceptible to root pathogens. In contrast, the “stem pitting” (SP) disease caused by CTV results from aberrant phloem development, resulting in visible pits in the wood. This disease does not cause tree death, but substantially reduces vigor and yield of sweet orange and grapefruit trees resulting in chronic yield reductions and high cumulative economic losses. SP is not specific to any particular rootstock. The third CTV-induced syndrome, “seedling yellows” (SY) is characterized by stunting and leaf chlorosis when small sour orange, grapefruit, or lemon trees become infected (Fraser, 1952). Other varieties do not develop these symptoms. Sometimes, the stunting and chlorosis is so severe that there is a complete cessation of growth. Remarkably, the fourth CTV syndrome in citrus is a complete lack of symptoms in almost all varieties, even including the decline-sensitive sweet orange/sour orange rootstock combination, even though the virus multiplies to high titers. For instance, most citrus trees in Florida are infected with mild isolates that cause no disease symptoms.

## Stem pitting

Interference with differentiation or development results in numerous phenotypes induced by viruses. Lack of chloroplast development that causes chlorosis is probably the most common virus-induced symptom. The reduced photosynthesis causes reduced growth. SP is a common virus-induced phenotype of perennial woody plants that results from interference with stem growth. In healthy and in normally developed areas of infected trees, the cambium, which is between the phloem and xylem, divides and differentiates in opposite horizontal directions producing new xylem on the inward side and new phloem on the bark side resulting in increased girth of the tree trunk and branches. Stem pits develop in areas where development is disrupted. The surrounding areas grow normally leaving the disrupted areas as indented areas or pits. A range of different viruses distributed throughout the plant virus taxon induce SP in a range of plant species, including numerous Prunus species, apples, vinifera grapevines, citrus, and avocado, usually resulting in a slow decline of growth and poor yields. Although this disease phenotype is common in virus-infected perennial woody plants, there is little understanding of the processes that cause the stem pits.

CTV causes SP diseases that greatly limit production in many citrus industries around the world and areas that do not have isolates that cause this disease spend considerable effort to keep it out (Bar-Joseph et al., 1989; Moreno et al., 2008). Affected trees with severe SP grow poorly, lack vigor, and yield small, unmarketable fruit. Acid limes are very susceptible, sweet oranges and grapefruit also are susceptible, while mandarins are more tolerant. The disease is not associated with scion/rootstock interactions and pitting can occur on either scion or rootstock or both. Citrus production areas in which severe SP isolates are endemic can be productive only by using mild strain cross protection or by not growing susceptible varieties.

**Figure 3:**
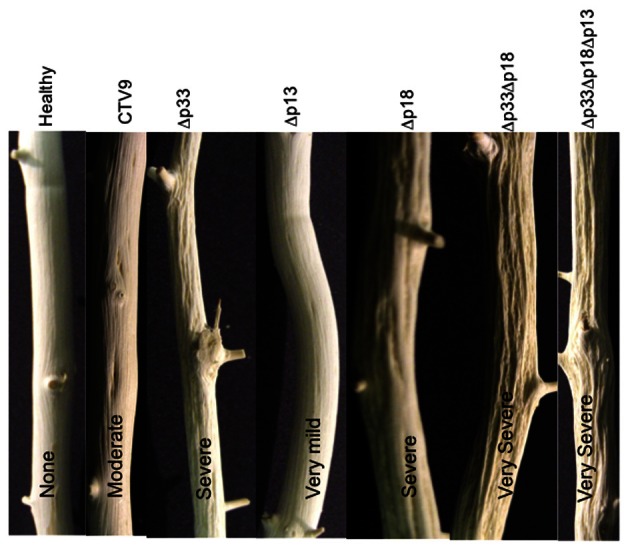
**Stems of *Citrus macrophylla* infected with mutants of CTV with all combinations of deletions of the p33, p18, and p13 genes showing different degrees of stem pitting**.

Brlansky et al. (2002) found that the formation of pits by CTV apparently is due to the inhibition of production of new xylem in the localized sites affected. The normally developing surrounding areas continue to grow leaving a depression or pit at the affected area. We examined the association of CTV with the formation of stem pits by tagging GFP to the mutants that induced this symptom (Tatineni and Dawson, 2012). Since CTV has three non-conserved genes (p33, p18, and p13) that are not required for systemic infection of some species of citrus (Tatineni et al., 2008), this allowed us to examine the effect of deletions of these genes on symptom phenotypes. In the most susceptible experimental host, *Citrus macrophylla*, the full-length virus causes only very mild SP symptoms. Surprisingly, we found that certain deletion combinations (p33 and p18 and/or p13) induced greatly increased SP, while other combinations (p13 or p13 plus p18) resulted in reduced SP (Figure 3).

One unexpected result was that in severely pitted areas, GFP fluorescence as a marker of virus replication was observed in regions normally made up of mature xylem or wood (Tatineni and Dawson, 2012). CTV was found in a group of cells that appeared to be on the woody side of the vascular system. In normally developing trees, most of the cells in this area differentiate into tracheary elements, which essentially consists of dead cells with thick walls connected into vessels for water transport. Interspersed in this area are live ray cells that transport nutrients from the phloem. In the full-length virus-infected trees, the fluorescence of GFP always was limited to the phloem ring outside of the cambium layer. However, increased SP was associated with virus-infected cells in areas not normally infected. Since CTV only multiplies and produces GFP in living cells and free GFP was not found in non-infected adjacent cells (Folimonov et al., 2007), it would not be expected that the virus could produce GFP in mature xylem cells without virus replication nor could GFP made in other cells accumulate in xylem. However, it should be kept in mind that this is a process that occurs over a period of time and the stem increases in girth as the plant grows in the presence of the viral infection. These results suggest that the process of forming a stem pit is not only the lack of producing new xylem in the affected area resulting in a depression in the wood, but also is affecting development and causing cells within the pitted area to continue living and to be susceptible to CTV invasion and replication.

Previously, it was expected that a specific CTV gene product induced SP, and further this product could be used to identify isolates of virus that would cause this disease. In contrast, removal of sequences induced SP. How could deletions in CTV induce severe SP?

Deletion of the p13 ORF tended to be correlated with reduced SP. Thus, deletion mutants that retained the p13 gene (deletion of p33, p18, or p33 plus p18) tended to have the most SP, which might suggest that the p13 gene product was involved in induction of stem pits. However, the triple deletion mutant, which did not have the p13 gene, induced severe SP, demonstrating that interpretation is not so simple. In contrast, increased SP generally was associated with deletion of the p33 ORF. Mutants with the absence of the p33 ORF (deletion of the p33 plus the p18 ORFs, and the p33 plus the p18 and p13 ORFs) induced severe SP. Thus, mutants retaining the p33 gene (deletions of p13, p18, or p13 plus p18) had the least amounts of SP. These results suggest that the presence of the p33 protein could be correlated with reduced SP (its absence increases it). However, the mutant with the deletion of the p18 ORF (p33 and p13 retained) induced moderate SP. Overall, the production of stem pits or no stem pits appears to be related more to a balance between expression of the p33 and p13 and possibly p18 genes (Tatineni and Dawson, 2012).

In general, deletions in CTV resulted in a substantial increase in the SP disease of citrus. Yet, there are different phenotypes of SP. Some trees have large stem pits that are readily visible in tree trunks and limbs without removing the bark. Other trees exhibit “cheesy bark” SP, which is a high density of very small pits. There is a continuum of levels in between. Some cause rapid decline of tree growth and yield, while others cause little damage to the tree. Additionally, there is the extreme specificity between virus isolates and different citrus species and varieties. It should be noted that most of the other hosts examined did not form stem pits when infected with these mutants (Tatineni et al., 2008, 2011; Tatineni and Dawson, 2012). There is no reason to think that all of the different SP phenotypes in different citrus hosts would be caused by the same virus-host interactions.

## Seedling yellows

The SY reaction is specific to only certain citrus hosts of CTV during the seedling stage, such as lemons, sour orange, and grapefruit, indicating that there are specific host factors involved in its expression in addition to isolate-specific viral factors. Mild SY symptoms are characterized by slight yellowing of new leaves and slight reduction in growth. Severe SY results in production of very small new leaves following infection. These leaves can be so chlorotic as to be almost white. The plants generally grow no more. Occasionally plants recover from SY and produce a new flush with normal leaves (Wallace and Drake, 1972).

In Florida, the decline isolate of CTV, T36, induces SY, whereas the widely distributed mild isolate, T30, does not. To delimit the viral sequences associated with the SY syndrome, we created a number of T36/T30 hybrids by substituting T30 sequences into different regions of the 3′ half of the genome of T36 (Albiach-Martí et al., 2010). Since T36 induces SY symptoms, the objective was to identify sequences that when substituted by T30 sequences would result in not inducing SY. T36/T30 hybrids were used to inoculate sour orange and grapefruit seedlings. Most of the T30/T36 hybrid constructs continued to induce SY symptoms identical to those of T36; however, two hybrids with T30 substitutions of the 3′-most gene (p23) and the 3′ NTR (nucleotides 18,394–19,296) failed to induce SY. This result suggested that the corresponding region of T36 (p23 to the 3′ end) was the determinant of this phenotype (Albiach-Martí et al., 2010).

## Decline

Historically, decline has been the most devastating disease caused by CTV. It caused the death of almost 100 million trees, largely in the Americas early in the last century (Bar-Joseph et al., 1989; Moreno et al., 2008). It is a man-made disease based on propagation of sweet orange, grapefruit, and mandarins on the sour orange rootstock. This process was largely due to root rot caused by oomycetes of the genus *Phytophthora*. When growers learned that sour orange was resistant to this disease, industries were converted to this rootstock. This set up a disaster when CTV was brought into the areas in infected propagation materials. Remarkably, the virus does not cause decline in sour orange trees on their own roots, but causes an incompatibility at the graft union that kills other varieties grafted onto this rootstock. Sometimes death can occur in as short a period as a few days, providing the classic picture of a dead tree full of fruit but with no leaves. Yet, the disease easily can be controlled by using alternative rootstocks. However, there are soils in which all other rootstock choices are less desirable in terms of fruit quality and yield.

Decline has been the major problem caused by CTV in Florida because fortunately severe stem-pitting isolates have been kept out so far. Yet, there are soils in which other all other rootstock choices are deficient compared to the sour orange rootstock. Thus, one of our major projects has been to find a way to allow growers to use the sour orange rootstock. Florida has two predominant strains of CTV, a decline strain (T36) and a mild strain (T30). Remarkably the T30 strain does not induce decline. In comparing the two strains, it appears that T36 contains determinants that induce decline that T30 does not have. In an attempt to identify the decline determinants, we have made hybrids in T36 in which T36 sequences are removed and substituted by T30 sequences, similar to the mapping exercise to identify SY determinants. However, this project has lingered due to our inability to assay for decline in the greenhouse with small trees. Under these conditions, sweet orange on sour orange rootstocks grow normally. Apparently, the small trees replace phloem as fast as the virus causes damage to it. We now have a field test on which we await results.

The potential control strategy is to use cross protection (superinfection exclusion: see Folimonova in this series) to protect trees on the sour orange rootstock. Since T36 and T30 are from different strains, T30 cannot be used to protect trees from T36 (Folimonova et al., 2009). Yet, a non-decline inducing isolate of the T36 strain could be used to protect against the endemic T36 isolates. But we have never been able to find a non-decline isolate of the T36 strain. However, perhaps such an isolate could be made. If we can identify sequences in T36 that induce decline, it should be possible to substitute those sequences from the T30 strain resulting in a T36 hybrid that does not cause decline. This hybrid could be inoculated to the commercial nursery trees on the sour orange rootstock to protect against decline.

## RNAi induction of symptoms?

Is the viral counter-attack of the host RNAi system a component of disease induction? It has been shown that ectopic expression of one of the CTV suppressors of RNAi, p23, induces virus-like symptoms (Ghorbel et al., 2001; Fagoaga et al., 2005; see Flores et al., 2013). In addition to intense vein clearing in leaves, transformed Mexican lime plants develop chlorotic pinpoints in leaves, stem necrosis, and collapse (Ghorbel et al., 2001), which usually are not symptoms associated with CTV infection. Transgenic sour orange plants expressing p23 also develop vein clearing, leaf deformation, defoliation, and shoot necrosis (Fagoaga et al., 2005). These transgene-induced symptoms differ from the virus-induced symptoms in sour orange. In transgenic limes, symptom severity parallels the accumulation levels of p23, regardless of the source or sequence of the transgene (Ghorbel et al., 2001; Fagoaga et al., 2005), whereas the symptom intensity in CTV-infected limes depends on the pathogenicity characteristics of the virus isolate. Yet, this difference in the host response could be related to the fact that, in transgenic plants, p23 is produced constitutively in most cells, whereas, in nature, p23 expression associated with virus infection is limited to phloem tissues.

In non-citrus species is has been shown that ectopic expression of viral suppressors of silencing alters mRNA expression levels and induces symptoms (Soitamo et al., 2011), therefore it may be speculated that suppression of host RNAi defenses alters that plant's small RNA regulatory pathways, resulting in symptom expression (Pacheco et al., 2012). It frequently has been observed that virus infections trigger an enrichment of both miRNA and passenger miRNA^*^ (Bazzini et al., 2011; Du et al., 2011; Hu et al., 2011). Virus infections have also been observed to initiate the expression of novel classes miRNA-like small RNAs (ml-sRNA) produced from the stem-loop precursors of conventional miRNAs (Hu et al., 2011). Changes in the expression of these small RNAs can lead to up or down regulation of their target mRNA (Pacheco et al., 2012). In virus-infected plants, changes in miRNA expression have been observed to up or down regulate genes involved in regulation of growth and cell differentiation (Hu et al., 2011; Pacheco et al., 2012). Changes in the accumulation patterns of sRNAs, including miRNAs, have been reported in CTV-infected citrus plants (Ruiz-Ruiz et al., 2011). Similarly, in citrus there are significant differences in the expression of miRNAs involved in transcription and hormone responses between healthy and CTV-infected plants, although their link to symptom expression remains unknown (Harper, unpublished). Thus, it appears likely that suppression of the host RNAi processes affects symptom production by CTV in at least some of its hosts, but remains an area of future research.

## Concluding remarks

CTV non-conserved genes apparently evolved to allow systemic infection of its hosts. These are genes involved in cell-to-cell and long-distance movement and in counter surveillance. Some are not needed for all hosts. These non-conserved genes can also be involved in induction of disease symptoms. A specific region was mapped to be involved in the SY syndrome. In contrast, deletion of genes was involved in induction of SP in *C. macrophylla*, apparently causing gene product ratios that induced abnormalities. In both cases, the symptoms resulted from an alteration of development. Interestingly, both of these disease symptoms are non-continuous. SY symptoms usually are transient. Plants respond only briefly and new growth does not exhibit the symptoms. SP is spatially sporadic. Some infected areas develop abnormally resulting in pits, but most other infected areas continue to develop normally.

Viruses evolve to survive in hosts with which they are presented. This involves acquiring and modifying genes to interact precisely with their hosts. A range of potential host species creates a bewildering array of selective factors; each species will differ to some degree in physiology, gene expression, metabolism, and antiviral defenses, and an isolate at an adaptive peak in one host may be less fit in another. The process of adaption to one host may also create the potential to cause disease in another. In citrus for example, most isolates are mild to asymptomatic in pomelo, mandarin, and citron (Garnsey et al., 1996), which are the three ancestral Citrus species (Nicolosi et al., 2000) and likely those in which CTV evolved. These same isolates, however, cause an array of symptoms on commercial citrus species, all of which are hybrids of the three ancestral species.

However, the results described above come from simple systems—a pure culture—a single strain of virus from a cDNA clone. Yet, most virus infections in the field are complex populations of mixtures of different strains and defective RNAs. Little is known concerning how these populations equilibrate and which components of the population interact with the host to elicit or prevent disease symptoms. Do components of the population complement to induce disease symptoms? Do some components counteract other components? The next frontier in plant virology is developing an understanding of populations.

### Conflict of interest statement

The authors declare that the research was conducted in the absence of any commercial or financial relationships that could be construed as a potential conflict of interest.
